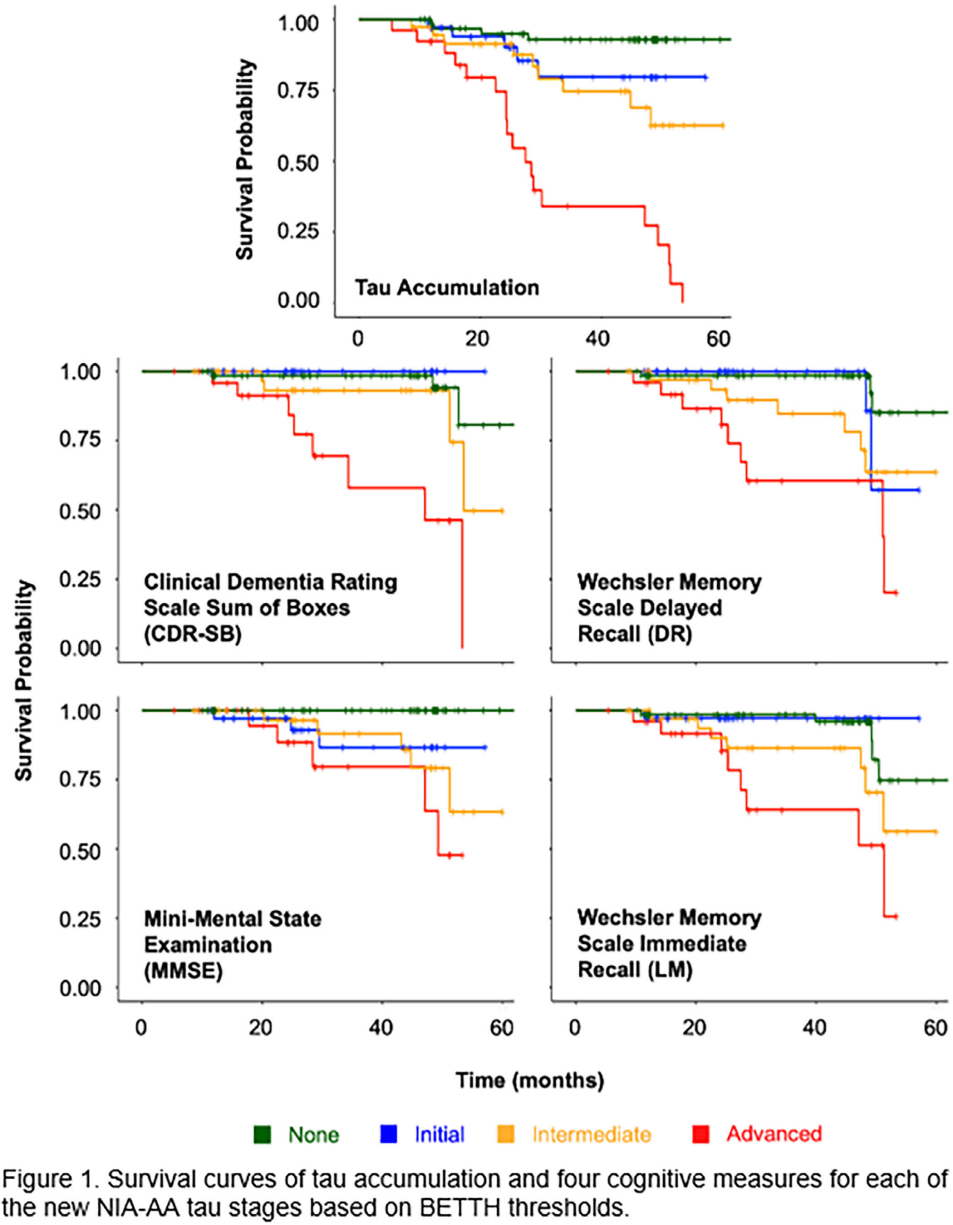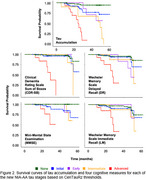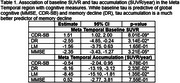# Implementation of NIA‐AA Multilevel Tau Staging for Predicting Tau Accumulation and Cognitive Decline in Non‐Demented Individuals

**DOI:** 10.1002/alz70856_105613

**Published:** 2026-01-08

**Authors:** Alexandra Gogola, Ann D Cohen, Beth E. Snitz, Davneet S Minhas, Dana L Tudorascu, Milos D. Ikonomovic, C. Elizabeth Shaaban, Vincent Dore, Cristy Matan, Cristy Matan, Alexander DelBene, Pierrick Bourgeat, Antoine Leuzy, Howard J Aizenstein, Oscar L Lopez, Brian J Lopresti, Victor L. Villemagne

**Affiliations:** ^1^ University of Pittsburgh, School of Medicine, Pittsburgh, PA, USA; ^2^ University of Pittsburgh School of Medicine, Pittsburgh, PA, USA; ^3^ University of Pittsburgh, Pittsburgh, PA, USA; ^4^ University of Pittsburgh Alzheimer's Disease Research Center (ADRC), Pittsburgh, PA, USA; ^5^ CSIRO, Melbourne, VIC, Australia; ^6^ University of Pittsubrgh, Pittsburgh, PA, USA; ^7^ CSIRO Health and Biosecurity, Australian E‐Health Research Centre, Brisbane, QLD, Australia; ^8^ Critical Path for Alzheimer's Disease (CPAD) Consortium, Critical Path institute, Tucson, AZ, USA

## Abstract

**Background:**

We evaluated the predictive performance of ^18^F‐flortaucipir (FTP) tau imaging within the NIA‐AA multilevel tau staging framework with respect to tau accumulation and cognitive decline in non‐demented individuals. We also tested the relationships of cognitive measures with baseline tau and tau accumulation.

**Methods:**

FTP scans from 213 non‐demented participants were processed and sampled in Statistical Parametric Mapping software (SPM), version 8, using CenTauR masks. Tau accumulation and cognitive decline associations were assessed longitudinally, with respect to two timepoints, their baseline and most recent evaluations, via survival analysis. Individuals were categorized into 4 groups reflecting the NIA‐AA imaging stages: Initial, with only b‐amyloid (Ab) pathology was present in PET; Early, with Ab pathology and tau pathology in the mesial temporal region; Intermediate, with moderate tau pathology in the meta temporal region; and Advanced, with high levels of tau in the meta temporal region. A “None” group reflecting no pathology was included as a control. Linear regressions were used to compare the longitudinal effects of either baseline tau (SUVR) or tau accumulation (SUVR/year) on cognitive decline.

**Results:**

While the two sets of thresholds yielded slightly different trajectories, both showed that when applying multiple levels of tau positivity, increasing stages of tau predicted both earlier tau accumulation and earlier cognitive decline. Linear regressions revealed that change in global measures of cognition (MMSE, CDR‐SB) were significantly associated with baseline tau, while decline in Delayed Recall (DR) was significantly associated with both baseline tau and tau accumulation, where tau accumulation had a greater influence in the model, and Immediate Recall (LM) decline was significantly only associated with tau accumulation.

**Conclusions:**

Implementing the multiple tau stages from the new NIA‐AA biological staging framework clearly predicts distinct patterns of tau accumulation and cognitive decline. While baseline tau is predictive of global cognitive decline, tau accumulation is a better predictor of memory decline. Future work is needed to determine how the thresholds utilized here compare to visual reads and to determine the suitability of these thresholds in differentiating trajectories of individuals with cognitive impairment.